# Evaluating Service-Learning with Older Adults on Undergraduate College Students During the COVID-19 Pandemic

**DOI:** 10.1093/geroni/igaa057.3455

**Published:** 2020-12-16

**Authors:** Sharon Merkin

**Affiliations:** UCLA Geffen School of Medicine, Los Angeles, California, United States

## Abstract

Introduction: Students in the Frontiers in Human Aging course at UCLA participate in service-learning (SL) with older adults. In 2020, completion of SL coincided with the outbreak of the novel coronavirus disease (COVID-19) pandemic. We evaluated the impact of SL on student attitudes on aging and community service in the context of the pandemic. Methods: Students were assigned to senior residential and daycare programs for 18-20 hours of SL. A retrospective pretest-posttest survey asked about attitudes and interests before and after SL and how the COVID-19 pandemic affected these perceptions; 73 (of 103) students responded. Mean differences before and after SL were tested and differences were assessed within groups reporting COVID-19 effects. Results: SL improved students’ attitudes and ability to engage with older adults, knowledge about aging concepts, interest in future work with older adults, attitudes on community service, social well-being and feelings of usefulness (p<0.001). There was no significant change in overall anxiety about aging (p=0.1), however, students showed increased anxiety about losing independence and finances when older (p<0.05). At least 50% of students reported that the COVID-19 pandemic increased their awareness of needs of older adults (81.9%) and decreased connection to their peers (50.7%); the impact of SL remained unchanged by these effects. Conclusion: Despite the overall benefits of SL, increased anxiety about aspects of aging suggests the need to address these concerns. While the COVID-19 pandemic did not seem to affect the impact of SL, this event did seem to influence perceptions about aging and social integration.

